# Primary pulmonary myxoid sarcoma in the interlobar fissure of the left lung lobe: a case report

**DOI:** 10.1186/s12890-024-03085-8

**Published:** 2024-07-03

**Authors:** Ting Xu, Li Wu, Hua Ye, Shuai Luo, Jinjing Wang

**Affiliations:** 1 Department of Pathology, Affiliated Hospital of Zunyi Medical University, Zun Yi City, Guizhou Province P.R. China; 2 Zhejiang Provincial People’s Hospital Bijie Hospital, Bijie City, Guizhou Province P.R. China

**Keywords:** Lung lobe interlobar fissure, Primary pulmonary myxoid sarcoma, Russell bodies, Diagnosis

## Abstract

**Background:**

Primary pulmonary myxoid sarcoma (PPMS) is a rare, low-grade malignant tumor, constituting approximately 0.2% of all lung tumors. Despite its rarity, PPMS possesses distinctive histological features and molecular alterations, notably the presence of EWSR1-CREB1 gene fusion. However, its precise tissue origin remains elusive, posing challenges in clinical diagnosis.

**Case demonstration:**

A 20-year-old male patient underwent a routine physical examination 6 months prior, revealing a pulmonary mass. Following surgical excision, microscopic evaluation unveiled predominantly short spindle-shaped tumor cells organized in a fascicular, beam-like, or reticular pattern. The stromal matrix exhibited abundant mucin, accompanied by lymphocytic and plasma cell infiltration, with Russell bodies evident in focal areas. Immunophenotypic profiling revealed positive expression of vimentin and epithelial membrane antigen in tumor cells, whereas smooth muscle actin and S-100, among others, were negative. Ki-67 proliferation index was approximately 5%. Subsequent second-generation sequencing identified the characteristic EWSR1-CREB1 gene fusion. The definitive pathological diagnosis established PPMS. The patient underwent no adjuvant chemotherapy or radiotherapy and remained recurrence-free during a 30-month follow-up period.

**Conclusions:**

We report a rare case of PPMS located within the left lung lobe interlobar fissure, featuring Russell body formation within the tumor stroma, a novel finding in PPMS. Furthermore, the histomorphological characteristics of this case highlight the diagnostic challenge it poses, as it may mimic inflammatory myofibroblastic tumor, extraskeletal myxoid chondrosarcoma, or hemangiopericytoma-like fibrous histiocytoma. Therefore, accurate diagnosis necessitates an integrated approach involving morphological, immunohistochemical, and molecular analyses.

## Background

Primary pulmonary myxoid sarcoma (PPMS) presents as a rare tumor with uncertain differentiation. Nicholson et al. first described PPMS in 1999 [1]. Subsequently, in 2011, Thway et al. coined the term PPMS based on its genetic profile characterized by EWSR1-CREB1 [2]. The World Health Organization recognized PPMS as an interlobar tumor for the first time in 2015 [3, 4]. Despite its classification as an interlobar-origin tumor and the identification of EWSR1-CREB1 gene fusion through various molecular techniques such as fluorescence in situ hybridization (FISH) testing, reverse transcription-polymerase chain reaction (RT-PCR), or second-generation sequencing, its tissue origin remains elusive. In the 2021 WHO classification of thoracic tumors, this neoplasm was designated as “PPMS with EWSR1-CREB1 Fusion” [5, 6]. Studies have revealed that PPMS often exhibits characteristic t(2;22) (q33;q12) chromosomal translocation, resulting in the oncogenic fusion gene EWSR1-CREB1 fusion. This fusion gene activates the transcription of target genes involved in cell proliferation and is detected in approximately 75% of cases. Histologically, PPMS shares features with mesenchymal malignant tumors, further complicating its tissue lineage determination. Immunohistochemical studies consistently demonstrate vimentin expression in most PPMS cases.

Literature (Table 1) suggests that the majority of PPMS cases are closely associated with bronchi but can also manifest in the interlobar fissure [7] including the case we present, which is unrelated to the bronchus and lacks infiltration into the lung parenchyma. Existing literature primarily comprises isolated case reports on PPMS. We present a case of PPMS incidentally discovered during a routine physical examination in the absence of clinical symptoms. Given its rarity, the bulk of our understanding of PPMS stems from isolated case reports.

**Table 1:** PPMS clinical pathological data

Case	Age(yr)	Sex	Smoking history	Clinical symptoms	Site/Tumor size (cm)	EWSR1-CREB1	Recurrence/Transfer	Follow-up(mouth)
1 [1]	27	F	Never	No symptom	RUU/4	NR	NED	180
2 [1]	43	F	20 years	Cough、Bronchitis	LUL/3.5	NR	NED	144
3 [2]	45	F	NR	Cough	RUL/1.5	EWSR1-CREB1 fusion	NED	12
4 [2]	36	F	NR	Neural symptom	L/NR	Neg	Cerebral metastasis	1.7
5 [2]	32	F	NR	Weight loss	RUL/NR	EWSR1-CREB1 fusion	NR	NR
6 [2]	28	M	Never	Cough, fever hemoptysis, Weight loss	LLL/2.8	EWSR1-CREB1 fusion	NR	36
7 [2]	67	M	Current	NS	LLL/2.8	EWSR1-CREB1 fusion	left renal metastasis	NR
8 [2]	68	F	NR	NR	RUL/2	NR	NR	NR
9 [2]	63	F	Never	hemoptysis	LUL/NR	EWSR1-CREB1 fusion	NR	48
10 [2]	51	M	NR	No symptom	RLL/2	EWSR1-CREB1 fusion	NR	NR
11 [3]	24	M	Never	No symptom	RLL/5	EWSR1-CREB1 fusion	NR	6
12 [4]	64	F	Never	Cough, fever	RUL/5.5	EWSR1-CREB1 fusion	pleural and bone metastases	24
13 [4]	27	M	Current	cough	RLL/5.0	EWSR1-CREB1 fusion	NED	29
14 [4]	45	M	Current	cough	LLL/3.0	EWSR1-CREB1 fusion	NED	24
15 [4]	43	M	Current	cough	RLL/2.0	EWSR1-CREB1 fusion	NED	4
16 [4]	23	M	Current	Cough, fever	RLL/3.0	EWSR1-CREB1 fusion	NDE	3
17 [4]	45	F	Never	cough	RUL/2.0	EWSR1translocation	NR	NR
18 [5]	40	M	NR	NR	RUL/2.2	EWSR1-CREB1 fusion	NED	15
19 [6]	12	F	Never	No symptom	LUL/10	EWSR1-CREB1 fusion	NR	36
20 [7]	31	M	NR	fever	RLL/2	EWSR1-CREB1 fusion	NED	12
21 [8]	80	F	NR	NR	in bronchus	EWSR1-CREB1 fusion	NR	23
22 [9]	45	F	Never	No symptom	RUL/2	Neg	NED	0
23 [10]	44	M	Never	No symptom	LUL/2	Neg	NED	68
24 [11]	48	M	Current	COPD	R AND L/14	EWSR1-CREB1 fusion	Death followed a few months after the brain metastases	17
25 [12]	64	M	Current	Cough, blood-stained sputum	RLL/15	EWSR1-CREB1 fusion	NED	NR
26 [13]	31	M	NR	NR	NR	EWSR1-CREB1 fusion	NED	NR
27 [14]	29	F	Never	NR	left interlobular fissure/	EWSR1-CREB1 fusion	NED	NR
28 [15]	39	F	NR	NR	LUL/1.6	EWSR1translocation	NED	NR
29 [15]	67	F	NR	NR	L/NR	EWSR1translocation	NED	NR
30 [15]	57	F	NR	NR	R/2.2	EWSR1-CREB1 fusion	NED	NR
31 [15]	41	M	NR	NR	RLL/5.4	EWSR1-CREB1 fusion	NED	48
32 [15]	33	F	NR	NR	RUL/1.7	EWSR1translocation	NR	NR
33 [16]	75	NR	Current	immunocompromised	NR/4.7	EWSR1translocation	NR	NR
34 [17]	30	M	NR	No symptom	LLL	EWSR1-CREB1 fusion	NED	48
35 [18]	41	F	NR	dyspnea	Right main bronchus	EWSR1-CREB1 fusion	NED	NR
our case	20	M	Never	No symptom	left interlobular fissure/4	EWSR1-CREB1 fusion	NED	30

F, Female; M, Male; NS, No Symptom; NR, Not Reported; NED, No Evidence Of Disease; RUL, Right Upper Lobe; RLL, Right Lower Lobe; LLL, Left Lower Lobe; LUL, Left Upper Lobe; L, Left Lung; R, Right lung; Adjacent to bronchus, endobronchial component involved (+) or not involved (-); Neg indicates negative no EWSR1 gene rearrangement or EWSR1 gene rearrangement

## Case demonstration

**Fig. 1:**
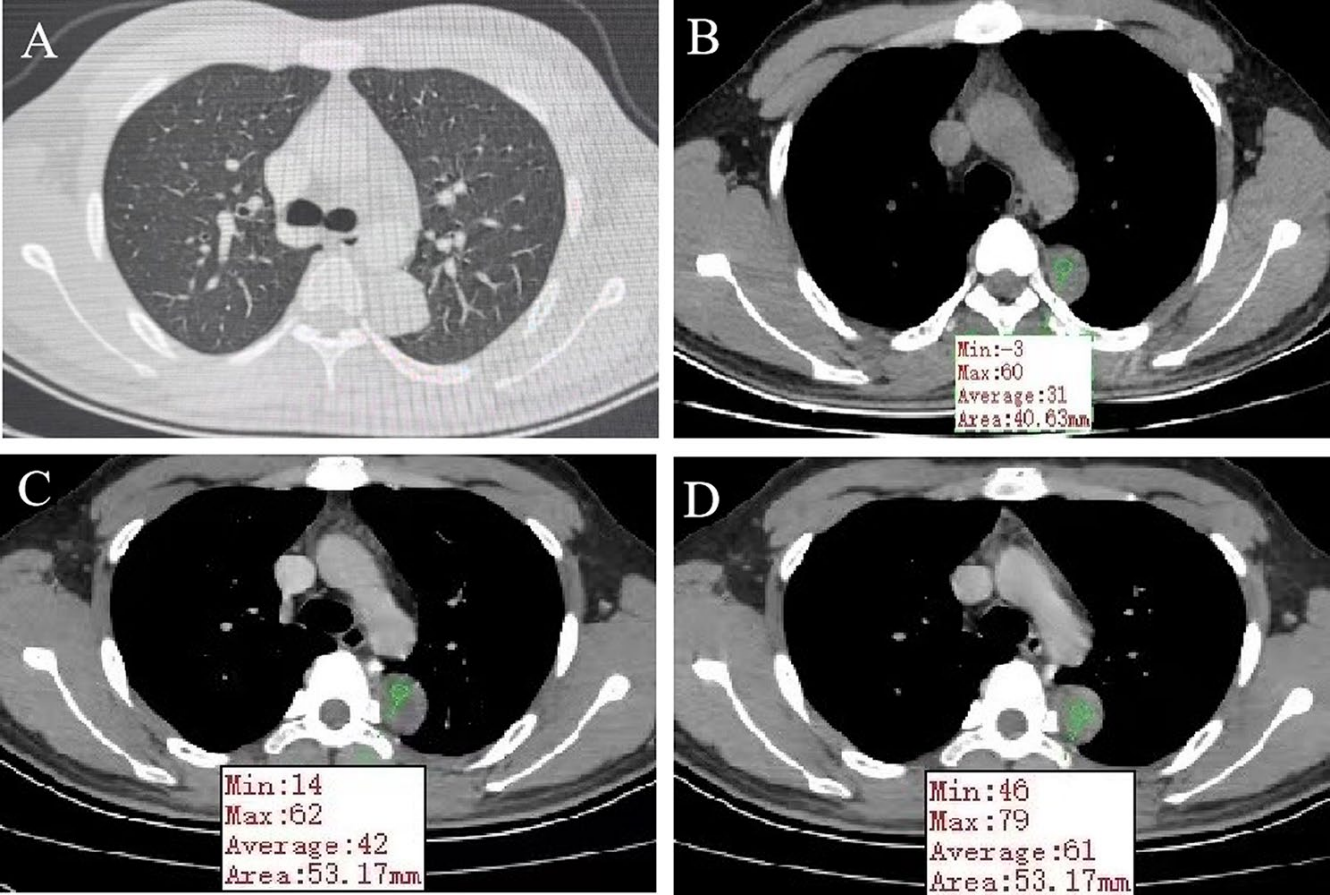
Computed tomography scans (**A**) revealed a mound-shaped soft tissue density shadow adjacent to the left posterior mediastinum at the fifth thoracic vertebra level, measuring approximately 4.0 × 2.8 × 2.1 cm, with well-defined borders and a broad base connecting to the inner left chest wall. Lung textures appeared normal, and bilateral pulmonary hila were not enlarged, with unobstructed airways bilaterally. Hilar lymph nodes and mediastinal lymph nodes were not enlarged. (**B**) Plain scan lesion CT value 31HU. (**C**) The CT value of arterial phase lesions was 42HU. (**D**) The CT value of the lesions in the venous phase was measured at 61 Hounsfield units (HU)

**Fig. 2:**
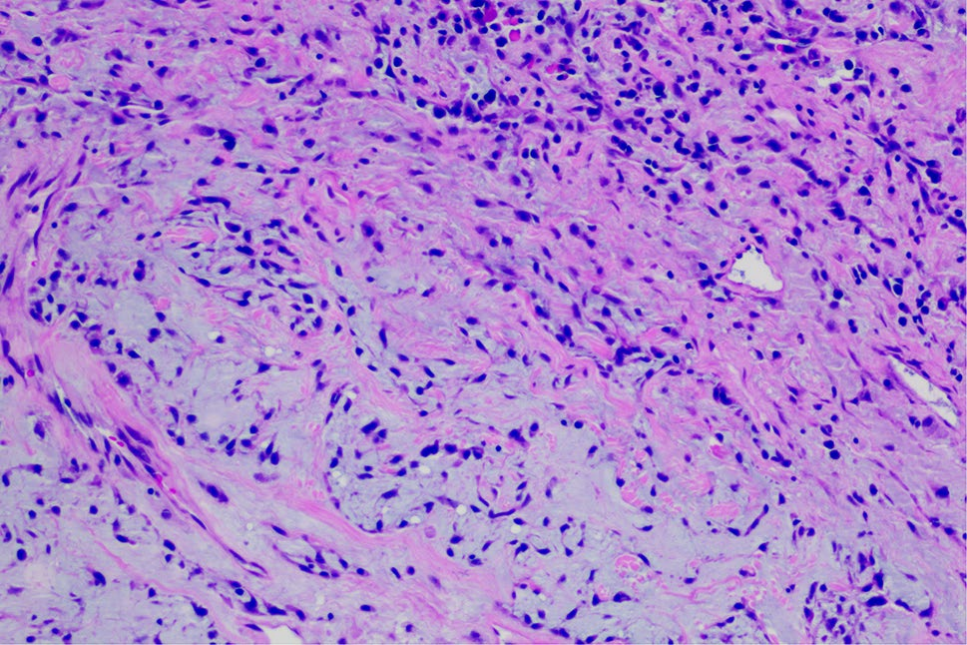
Microscopic examination, as depicted in this figure (HE 100X), reveals a nodular distribution of the tumor. Tumor cells appear as sparse spindle-shaped and short fusiform cells, with the stroma exhibiting mucoid and collagenous changes, accompanied by lymphocytic

**Fig. 3:**
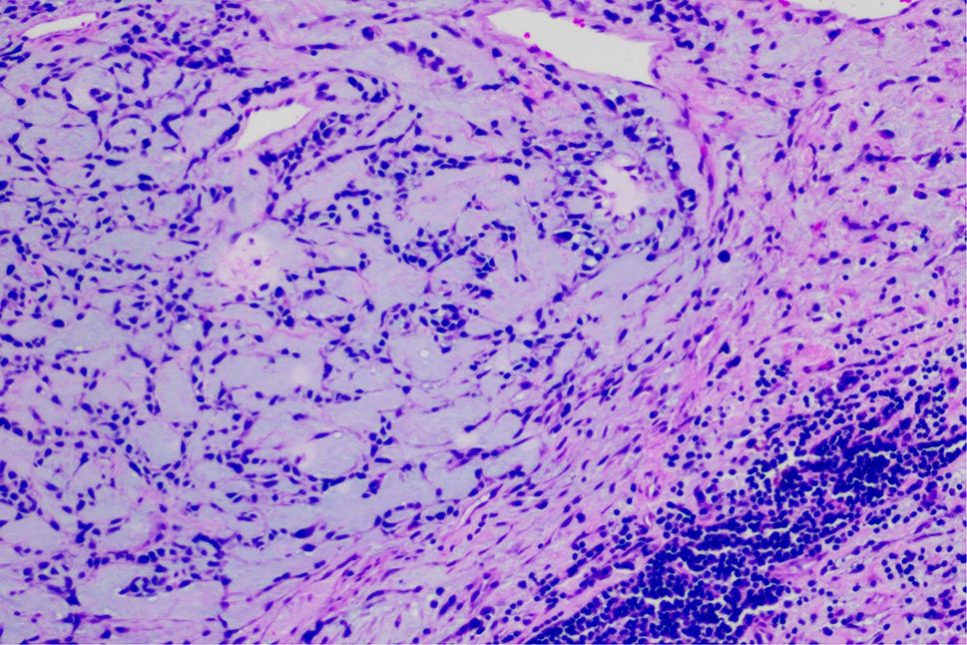
Microscopic examination, as depicted in this figure (HE 200X), reveals a nodular distribution of the tumor. Tumor cells appear as sparse spindle-shaped and short fusiform cells, with the stroma exhibiting mucoid and collagenous changes, accompanied by lymphocytic

**Fig. 4:**
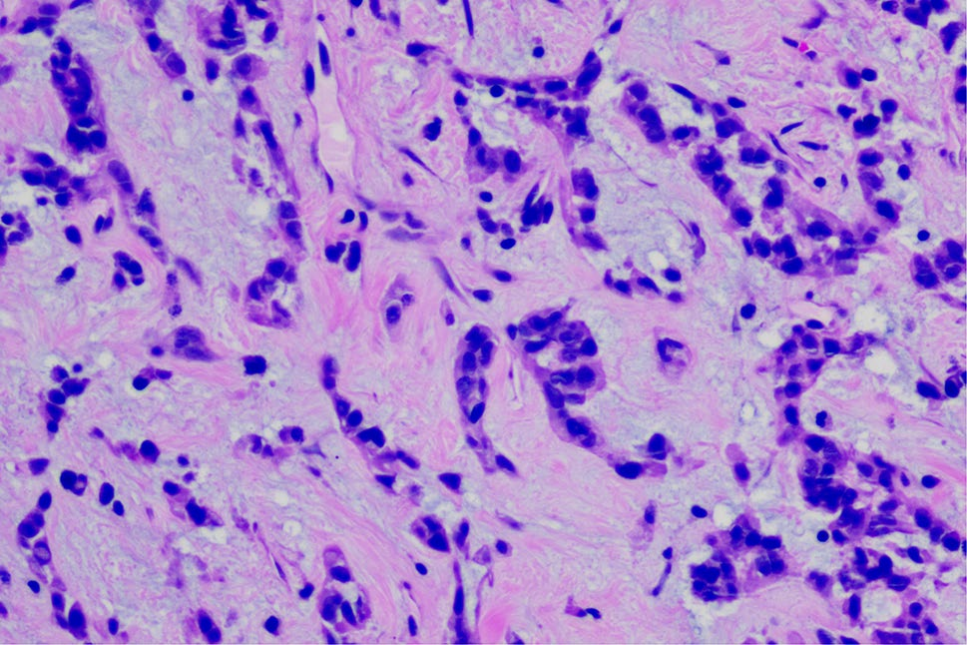
(HE 400X) At higher magnification, as shown in this figure (HE 400X), tumor cells appear short spindle-shaped, distributed within a mucoid background, displaying basophilic cytoplasm, fine chromatin, minimal cellular pleomorphism, and rare mitotic figures

**Fig. 5:**
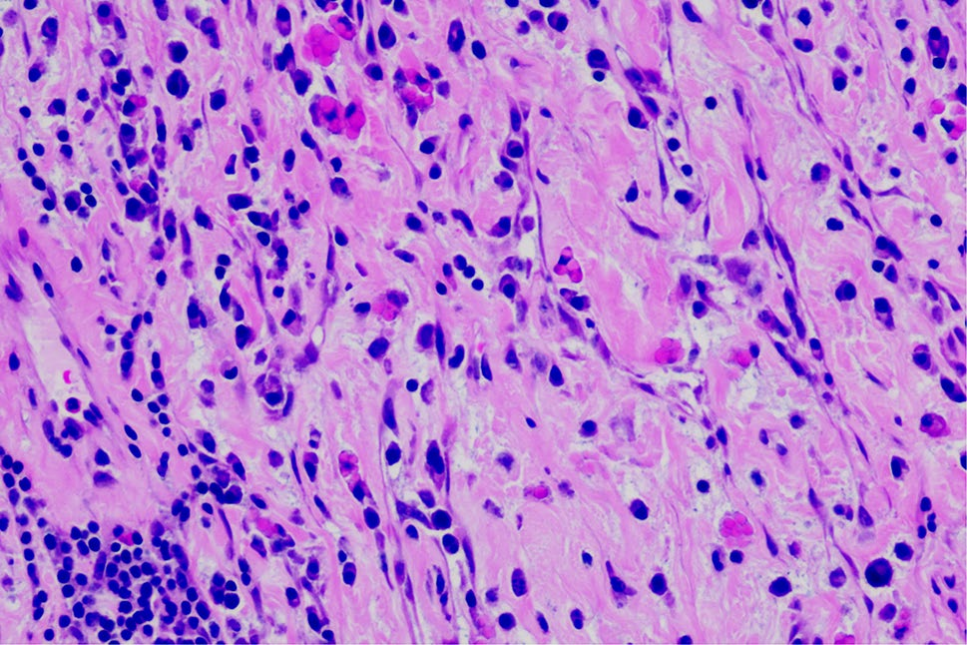
(HE 400X) displays foci of lymphocytic and plasma cell infiltration within the tumor stroma, with visible Russell body formation

**Fig. 6:**
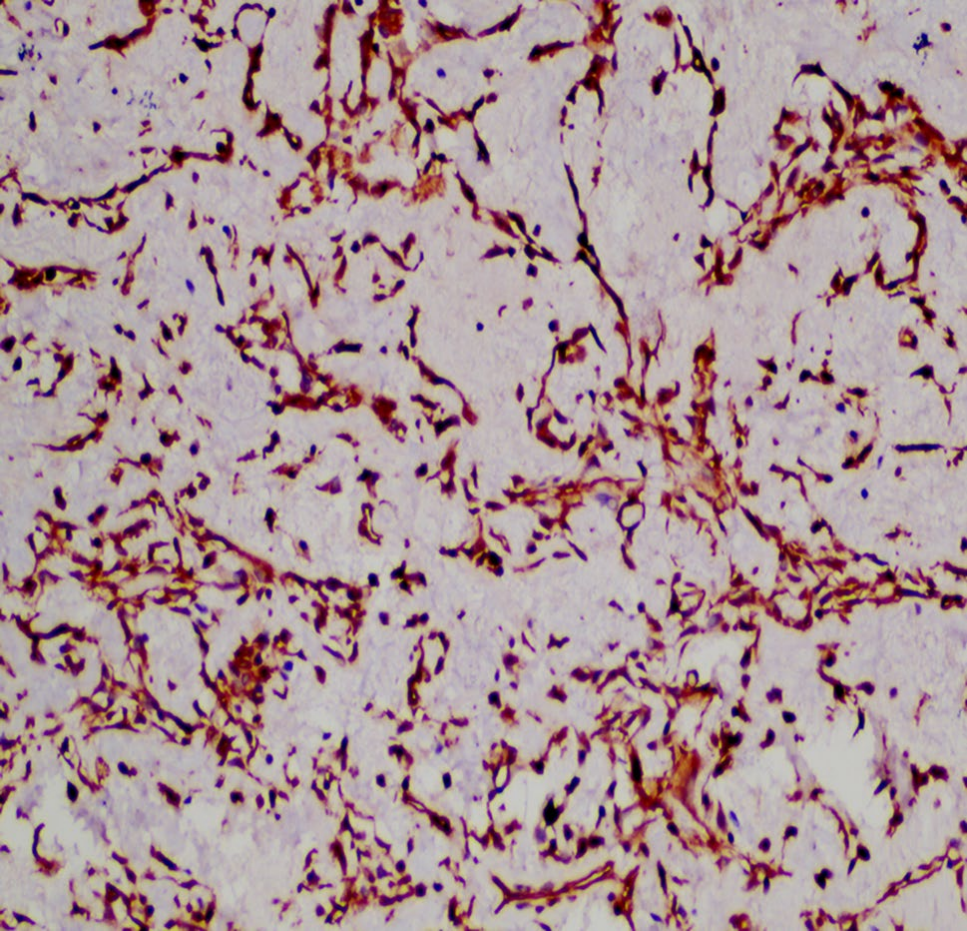
(IHC Envision 400X) shows positive vimentin immunohistochemical staining in tumor cells

**Fig. 7:**
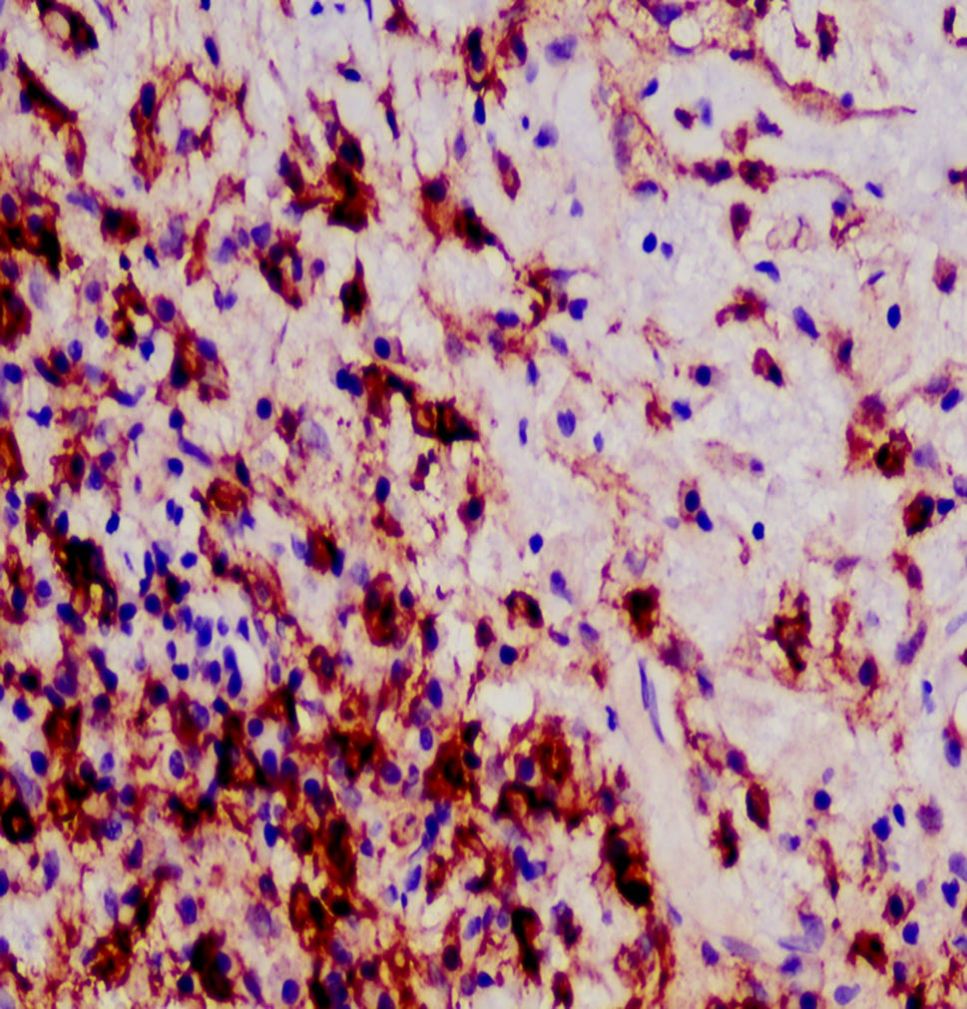
(IHC Envision 400X) reveals positive EMA immunohistochemical staining in tumor cells

A 20-year-old male patient was admitted following the discovery of a lung mass during a routine physical examination 6 months earlier. He presented without cough, sputum production, shortness of breath, or fever. Computed tomography scans (Fig. 1A) revealed a mound-shaped soft tissue density shadow adjacent to the left posterior mediastinum at the fifth thoracic vertebra level, measuring approximately 4.0 × 2.8 × 2.1 cm, with well-defined borders and a broad base connecting to the inner left chest wall. Lung textures appeared normal, and bilateral pulmonary hila were not enlarged, with unobstructed airways bilaterally. Hilar lymph nodes and mediastinal lymph nodes were not enlarged. Plain scan lesion CT value 31HU (Figs. 1B). The CT value of arterial phase lesions was 42HU(Figs. 1C). The CT value of the lesions in the venous phase was measured at 61 Hounsfield units (HU)(Figs. 1D). In response to the patient’s strong request for surgical intervention, thoracoscopic resection of the left pulmonary mass was performed. A circular mass measuring approximately 4 × 3 cm in size was identified in the posterior segment of the left interlobar fissure with well-defined boundaries and adhesion to the pleura during the procedure. The adhesion was carefully released and gradually separated along the surface of the mass. No residual mass or enlargement of hilar lymph nodes was observed. The tumor was excised and sent for pathological examination.Gross pathological examination revealed a single gray-white mass measuring 4.0 × 3.0 × 2.0 cm, featuring a smooth surface, complete capsule, and a mucoid appearance on cut sections, with minimal lung tissue at the edges.The frozen section revealed spindle cell proliferative lesions, with a significant infiltration of lymphocytes and plasma cells in the abundant mucous background. Distinguishing between benign and malignant was challenging. Under low microscopic magnification, the tumor exhibited nodular distribution with spindle-shaped and short fusiform tumor cells sparsely distributed in a background demonstrating mucoid and collagenous changes, accompanied by lymphocytic infiltration in the stroma (Figs. 2 and 3). Under high microscopic magnification (Fig. 4), the tumor cells were short spindle-shaped, dispersed within a mucoid background, displaying basophilic cytoplasm, fine chromatin, minimal cellular pleomorphism, rare mitotic figures, and lymphocytic and plasma cell infiltration regions, with certain areas exhibiting Russell body formation (Fig. 5). Immunohistochemical analysis revealed positive staining for vimentin (+) (Fig. 6), (epithelial membrane antigen) EMA (+) (Fig. 7), focal positivity for smooth muscle actin (SMA), and negativity for CK7, pan-CK, thyroid transcription factor 1 (TTF-1), CD34, carcinoembryonic antigen, S-100, anaplastic lymphoma kinase (ALK), with a Ki-67 index of 5% positive. Second-generation sequencing identified the gene fusion of EWSR1-CREB1. Through the integration of morphological, immunohistochemical, and molecular testings, the diagnosis was established as PPMS.

The patient was not treated with chemotherapy or radiotherapy after surgery, and had no respiratory symptoms such as cough, sputum, dyspnea, or fever. CT examination six months after surgery showed no tumor recurrence or metastasis. Up to now, 30 months of follow-up showed no recurrence, metastasis, respiratory symptoms and other clinical symptoms.

## Discussion

We comprehensively reviewed 35 previously reported cases of PPMS alongside the present case (Table 1). PPMS represents a low-grade microscopic malignant tumor, among patients aged 12–80 years (median age: 42 years), 18 females, 17 males, a female-to-male ratio of 1.06:1. The majority of literature depicts the mass as bronchus-related and potentially situated within the bronchus. However, we report a case where the mass was in the left lung lobe interlobar fissure without infiltration into the lung parenchyma. Most patients in the literature presented with respiratory symptoms [2, 7, 9–11, with occasional reports of chest pain and weight loss [2, 10]. Nevertheless, a minority of patients presented with no clinical symptoms at all [6, 14–16]. In our case, the patient was incidentally diagnosed during a routine physical examination and remained asymptomatic.

A total of 36 cases of primary progressive multiple sclerosis (PPMS) were documented in the existing literature, with 9 individuals having a history of smoking. The clinical manifestations of PPMS were non-specific, with 18 cases being asymptomatic, 10 cases presenting with cough, 3 cases exhibiting fever, and 2 cases experiencing weight loss. Neurological symptoms, hemoptysis, and immune deficiency were uncommon. All patients underwent surgical resection, with chemotherapy administered to 3 cases and radiotherapy to 1 case.Revised sentence: “This tumor is typically non-aggressive, and among the documented cases, one presented with brain metastases [2], another with renal metastases [2], a third with pleural and bone metastases [6], and a fourth with contralateral lung metastases [13, 16]. Despite the presence of metastases, the prognosis remained relatively favorable.

The tumor size varied from 1.5 to 15.0 cm (median size: 3.0 cm) (Table 1) and typically manifested as well-defined nodular masses. Grossly, the tumor displayed a gray-white or gray-yellow, gelatinous appearance on cut sections. Under low microscopic magnification, the tumor exhibited lobulated growth, featuring polygonal, spindle-shaped, or oval tumor cells arranged in a reticular or fascicular pattern within a mucoid matrix. The degree of cellular pleomorphism ranged from mild to marked, with the number of mitotic figures ranging from 0 to 32 per 10 high-power fields (HPFs), most commonly fewer than 5 per 10 HPFs.

A notable histological feature observed was varying degrees of interstitial myxoid degeneration. Chronic inflammatory cell infiltration, primarily comprising lymphocytes and plasma cells, was observed in most cases. Along with the typical histological morphology reported in the literature, our case exhibited a small number of Russell bodies. Russell bodies, characterized by individual eosinophilic globules within the cytoplasm of plasma cells [17, 18], can manifest in reactive conditions [14] as well as in neoplastic diseases [7, 19]. The significance of these inclusions in relation to diagnosis and prognosis awaits validation through extensive clinical data.When Russell bodies are detected, it is essential to differentiate them from inflammatory myofibroblastic tumors.

Immunohistochemical staining revealed near-universal expression of vimentin, with some cases showing focal weak expression of EMA and SMA. Conversely, markers such as AE1/AE3, S-100, desmin, CD34, ALK, and TTF-1 were consistently negative, and the Ki-67 proliferation index was generally low [2, 14, 16]. In our case, the Ki-67 proliferation index was approximately 5%, consistent with existing literature.

This tumor is characterized by the t(2;22)(q33;q12) translocation, encoding the fusion gene EWSR1-CREB1. In most cases, confirmation of the EWSR1-CREB1 gene fusion was achieved through RT-PCR, FISH, or second-generation sequencing. In our reported case, the presence of the EWSR1-CREB1 gene fusion was confirmed via next-generation sequencing.The presence of this gene fusion could potentially be targeted for pharmaceutical intervention, providing a basis for further investigation into the management of PPMS.

In the diagnostic process, differentiation from several other tumors is crucial:

1. Distinguishing from extraskeletal myxoid chondrosarcoma: Extraskeletal myxoid chondrosarcoma typically spans a wide age range (5–90 years) and commonly arises in the deep soft tissues near the proximal end of the lower limbs. It can metastasize to the lungs and histologically presents as lobulated structures composed of oval to short spindle-shaped cells arranged in a reticular, small cluster, or mesh-like pattern within a mucoid matrix [20, 21]. Morphologically, it can be challenging to differentiate from PPMS. However, considering patient history and molecular testing aids in differentiation. PPMS often exhibits EWSR1-CREB1 gene fusion [12, 22], whereas approximately 75% of extraskeletal myxoid chondrosarcomas display EWSR1-NR4A3 gene fusion.
2. Distinguishing from inflammatory myofibroblastic tumor (IMT): IMT is frequently observed in children and young individuals, with the lungs being a common site of involvement. Histologically, it manifests as myofibroblastic cells within a mucoid background, accompanied by abundant plasma cells, lymphocytes, or eosinophils. Russell body formation may also occur. IMT often expresses SMA, vimentin, and ALK, with molecular genetics revealing ALK-TPM3, RANBP2-ALK, and RRBP1-ALK gene fusions [23, 24].
3. Distinguishing from angiomatoid fibrous histiocytoma (AFH): AFH predominantly occurs in children and young individuals, primarily in the deep dermis and subcutaneous tissues of the extremities. Histologically, it presents as a proliferation of histiocytic/spindle cells in sheets or short bundles, accompanied by dense lymphoplasmacytic infiltration. AFH typically exhibits immunohistochemical positivity for vimentin and EMA, with other fusion genes such as EWSR1-ATF1 and FUS-ATF1 [25].
4. Distinguishing from soft tissue myoepithelial tumor: Soft tissue myoepithelial tumors lack a distinct age peak and commonly occur between 40 and 45 years, affecting both sexes equally. Morphologically resembling myoepitheliomas arising from salivary glands, these tumors present as multiple nodular or lobulated masses with various types of mucoid, chondroid, or clear interstitium, growing in a reticular or small beam-like pattern within a mucoid background. Immunohistochemistry typically demonstrates positivity for CK, p63, SMA, calponin, and S-100 protein, all of which are negative in PPMS. Myoepitheliomas exhibit fusion genes such as EWRS1-ZNF444, EWRS1-PBX1, and EWRS1-ATF1 [26].
5. Distinguishing from primary pulmonary meningioma: Extremely rare, primary pulmonary meningioma displays small to medium-sized spindle or epithelioid cell cords arranged in a bundle-like pattern, with eosinophilic cytoplasm and rich mucoid or vesicle-like matrix within round nuclei. Immunohistochemistry typically shows positivity for EMA and vimentin [27, 28] but lacks the t(2;22)(q33;q12) chromosomal translocation.

The previously reported cases of primary pulmonary mucoepidermoid carcinoma (PPMS) were predominantly managed through surgical intervention. Out of the 36 documented cases, only 4 exhibited metastasis: one with brain metastasis [2], one with renal metastasis [2], one with pleural and bone metastasis [6], and one with contralateral lung metastasis [13, 16]. Despite the presence of metastasis, all patients succumbed to the disease, although the prognosis was relatively favorable. Further research is required to explore alternative treatments. The patient was monitored for 30 months without receiving chemotherapy or radiotherapy, and no recurrence or metastasis occurred.

## Conclusion

In conclusion, PPMS represents a tumor with an elusive origin, predominantly affecting female patients, with onset typically occurring in young to middle-aged individuals. Classified as a low-grade malignant or malignancy of undefined potential, PPMS commonly presents with coughing and hemoptysis as initial symptoms. Microscopically, the tumor exhibits abundant short spindle-shaped cells distributed within a mucoid matrix. Immunohistochemistry lacks specific marker expression, whereas the presence of EWSR1-CREB1 gene fusion serves as a typical molecular pathological characteristic observed in approximately 75% of cases. Surgical resection constitutes the primary treatment modality, resulting in generally favorable prognoses for patients with PPMS. Among 35 previously reported cases, metastasis occurred in 4 cases, with only 1 death, whereas the remaining patients survived.

We present the first documented case of PPMS occurring in the left lung lobe interlobar fissure, accompanied by the presence of Russell bodies in the stromal area.The presence of Russell bodies in this case report necessitates validation through extensive clinicopathological data to determine its correlation with the diagnosis and prognosis of the disease.

## Data Availability

All the data regarding the findings are available within the manuscript.

## Abbreviations

CT: Computed Tomography
PPMS: Primary Pulmonary Myxoid Sarcoma
RT-PCR: Reverse Transcription-Polymerase Chain Reaction
